# The Relationship Between Ankle-Brachial Index and Number of Involved Coronaries in Patients with Stable Angina

**Published:** 2010

**Authors:** Masoumeh Sadeghi, Aliakbar Tavasoli, Hamidreza Roohafza, Nizal Sarrafzadegan

**Affiliations:** 1 Associated Professor of Cardiology, Isfahan Cardiovascular Research Center, Isfahan University of Medical Sciences, Isfahan, Iran; 2 Associated Professor of Cardiology, School of Medicine, Isfahan University of Medical Sciences, Isfahan, Iran; 3 Psychiatrist of Isfahan Cardiovascular Research Center, Isfahan University of Medical Sciences, Isfahan, Iran; 4 Professor of Cardiology, Isfahan Cardiovascular Research Center, Isfahan University of Medical Sciences, Isfahan, Iran

**Keywords:** Ankle to brachial index, Coronary involvement, Stable angina

## Abstract

**BACKGROUND:**

Atherosclerosis is the commonest cause of vascular disease which can involve peripheral and/or cardiac vessels. This study was conducted to evaluate the possible link between Ankle-Brachial Index (ABI) and coronary vessel involvement in patients with stable angina.

**METHODS:**

This cross-sectional study was conducted in 2008 on 120 individuals who were hospitalized in Chamran Heart Center and underwent coronary angiography. A questionnaire was completed to obtain demographic information, history of previous heart disease and smoking. Body height and weight, as blood pressure on hand and foot were measured. The patients underwent angiography and the extent of coronary involvement (> 75%) was determined. After12-14-hour of fasting, blood sugar was obtained to measure total cholesterol, triglyceride, low-density lipoprotein cholesterol (LDL-C) and high-density lipoprotein cholesterol (HDL-C). The Ankle Brachial Pressure Index (ABI) was calculated as the ratio of the blood pressure in the ankles to the blood pressure in the arms. The data were analyzed by SPSS-15 using ANOVA, T-Student test, Spearman's rank correlation coefficient, and discriminant analysis.

**RESULTS:**

Samples were 46 women (38.33%) and 74 men (61.67%) with a mean age of 55.50 ± 10.49. Mean and SD of ABI in men and women was 0.72 ± 0.20 and 0.80 ± 0.19 with no significant difference (P=0.012). The correlation between ABI and extent of coronary involvement was 0.47 (P < 0.0001). The group with lower ABI had the highest levels of coronary involvement (triple vessel, P < 0.05).

**CONCLUSION:**

ABI had a significant relationship with the degree of coronary involvement and a significant predictive value. Therefore ABI seems to be a reliable indicator of high coronary risk.

## Introduction

Coronary artery disease (CAD) affects 20% of the Iranian population, accounting for the largest number of deaths in Iran.^1^ A similar paradigm has been demonstrated in other countries too. Forty percent of mortalities in the US are CAD-related.^2^ Atherosclerosis is the commonest cause of coronary disease; more than 90% of heart attacks are due to atherosclerosis.^3^

Atherosclerosis can affect all body vessels. Peripheral vessel atherosclerosis may exist with or without cardiac vessel involvement.^4^ Risk factors related to coronary and peripheral vessels are similar, including aging, smoking, hypertension, dyslipidemia, and diabetes mellitus.^5^

Peripheral and coronary angiography is the standard method of detecting vessel involvement; however, this is an invasive procedure with many complications. In addition to angiography, non-invasive procedures e.g. ultrasonography and measurement of the Ankle-Brachial Index (ABI) can be used to detect peripheral vessel disease.^6^

The relationship between coronary and peripheral vessel involvement via ABI measurement is well known in the elderly, but is unclear in individuals with moderate risk and stable angina without symptomatic peripheral vessel disease.^7^–^9^ Based on the prevalence of peripheral vessel disease in various countries, the predictive value of peripheral and cardiac vessel involvement has been expressed differently.^9^ In various studies, the relationship between involvement of these vessels is different.^10^

The prevalence of peripheral vessel disease is different in various communities, ranging from 4.6 to 29%.^11^–^14^ One study reported a sensitivity of 34.3% and specificity of 87% for ABI in predicting coronary involvement based on peripheral vessel disease.^15^ Another study demonstrated low ABI to be associated with greater involvement of 3 or 4 coronary vessels.^16^ Another study reported the prevalence of peripheral vessel disease in South India to be significantly lower than in Western countries.^17^ Moreover, most patients with peripheral vessel disease in India were found to have normal coronaries.^18^ Given the high prevalence of CAD, varying prevalence of peripheral and coronary vessel diseases in different societies, varying predictive value of ABI, inexpensiveness, ease and accessibility of ultrasonographic measurement, this study was conducted to examine the relationship between peripheral and coronary vessel involvement.

## Materials and Methods

This cross-sectional (descriptive analytical) study was conducted at Isfahan Chamran Heart Center in 2008 on 120 individuals undergoing coronary angiography following presentation with typical chest pain (retrosternal pain worsening with exertion/subsiding with rest). Simple sampling method was used. Questionnaires obtaining demographic information including age/sex, as well as history of hypertension, smoking, diabetes mellitus, dyslipidemia and ischemic heart disease were completed for patients. Patients who were smoking any number of cigarettes daily were considered as smokers.^19^

A trained person measured the patients' body weight/height when they were lightly dressed and had no shoes on. Blood pressure in the ankles and the arms was measured. Systolic and/or diastolic pressure greater than 140 and 90 mmHg respectively, or use of antihypertensive medications was considered as hypertension.^20^ A Madeco Biderectional Smatdop 30 ultrasonography device (made in Japan) and a pneumatic cuff was used by a cardiologist to measure the brachial artery and ankle pressure waves. Ankle Brachial Pressure Index (ABI) was calculated as the ratio of the blood pressure in ankles to the blood pressure in the arms.

After 12-14 hours fasting, blood samples were obtained. Measurement of total cholesterol, triglyceride and blood glucose was performed by an autoanalyzer using the spectrophotometry method. HDL-C was measured after heparin-manganese sedimentation.^21^ When triglyceride concentration was below 400 mg/dl LDL-C was calculated with the Friedwald formula and when it exceeded 400 mg/dl it was determined by an ELAN2000 machine.^22^

Normal total cholesterol, triglyceride and LDL-C levels were considered below 240 mg/dl, 200 mg/dl and 160 mg/dl, respectively. Normal HDL-C for men and women was considered > 40 mg/dl and > 50 mg/dl, respectively. Any deviation from these definitions and/or use of statins was defined as dyslipidemia.^23^ Fasting blood glucose equal to or greater than 126 mg/dl or 2-hour blood glucose greater than 200 mg/dl and/or use of glucose lowering drugs was defined as diabetes.^24^

The degree of involvement was defined as follows:

1. No involvement: absence of coronary involvement.
2. Single-vessel involvement: coronary occlusion equal to or more than 75%.
3. Two-vessel involvement: coronary occlusion equal to or more than 75% in two coronaries or their main branch vessels. As left main is equal to double vessel disease, it categorized in this group.
4. Triple-vessel involvement: coronary occlusion equal to or more than 75% in all three coronaries.

ABI equal to or less than 9.0 was considered as peripheral vessel disease and ABI greater than 9.0 was considered as normal.^25^

The data were analyzed by SPSS_15_ using ANOVA, t-test, Spearman's rank correlation coefficient (to determine the correlation of ABI and degree of coronary involvement), and discriminated analysis (to assess the predictive value of ABI for coronary involvement).

## Results

This study was conducted on 120 patients consisting of 46 women (38.33%) and 74 men (61.67%) with a mean age of 55.50 ± 10.49 years. The patients had stable angina and had undergone coronary angiography and Doppler ultrasonography of limb vessels. The frequency of cigarette smoking at the time was 15% (8 patients); also 51% (61 patients) had family history of coronary disease. Of all, 27.5% (33 patients) were diabetic, 55.8% (67 patients) were hypertensive, and 70% (84 patients) were dyslipidemic.

In table 1, distribution of ABI mean and coronary artery involvement is shown.

**Table 1:** ABI mean and coronary artery involvement

ABI score	
Coronary artery involvement	Mean ± standard deviation
No involvement	0.882 ± 0.135
Single-vessel involvement	0.842 ± 0.036
Two-vessel involvement	0.687 ± 0.051
Triple-vessel involvement	0.663 ± 0.041

*P=0.001

Mean ABI was 0.72 ± 0.20 and 0.80 ± 0.19 in men and women, respectively, with a total average of 0.74 ± 0.20. T-test was used to compare mean of ABI in men and women; no significant difference was observed (P=0.12).

Also frequency distribution of coronary involvement in patients with stable angina was 28 (23.3%) in those with negative angiography results, 18 (15%) in patients with single vessel involvement, 21 (17.6%) in patients with two-vessel involvement, and 53 (44.1%) in patients with triple-vessel involvement.

Spearman's rank correlation coefficient was used to assess the correlation between ABI and degree of coronary involvement. The correlation coefficient measured 47.0 and P < 0.0001.

ANOVA was used to compare ABI in groups with varying degrees of coronary involvement; a significant difference was revealed (P=0.001). Turkey's test was used for further evaluation. Mean of ABI was different in patients with and without coronary involvement; the group with triple-vessel involvement was significantly different from other groups (P < 0.05).

As shown in Table 2, the frequency distribution of coronary involvement according to ABI is in the lower qualitative band (i.e.>9.0).

**Table 2:** frequency of patients according to ABI score

coronary artery involvement	No involvement	Single-vessel involvement	Two-vessel involvement	Triple-vessel involvement
ABI score				
Less than 0.9	16(17%)	15(15.1%)	17(18.9%)	34(49.1%)
More than 0.9	15(46.7%)	7(13.3%)	7(13.3%)	9(26.7%)

The group with lower ABI mostly had triple-vessel involvement and the above 9.0 group were mostly without coronary involvement (P < 0.05). To assess the predictive value of ABI, the discriminated test with a stepwise model was used to classify patients into four groups, namely without coronary involvement, single-vessel involvement, two-vessel involvement, and triple-vessel involvement.

In this test, ABI, age, sex, hypertension, hyperlipidemia, diabetes, current smoking history, and family history of coronary disease were entered into the model as independent variables and only ABI remained in the model. The difference between groups according to ABI as the predictive variable demonstrated a predictive value of 5.48% for ABI.

## Discussion

Based on our results, mean ABI score was not significantly different in men and women. We also found a link between ABI score in groups with various degrees of coronary involvement; the group with lower ABI had the highest degree of involvement (three vessels) and the group with ABI > 9.0 were mostly without involvement.

Various studies have highlighted the link between low ABI and peripheral vessel involvement. Guo et al studied the sensitivity and specificity of ABI in detecting stenosis of peripheral vessels and demonstrated an association between decreased ABI and degree of peripheral vessel involvement; the severest case of stenosis in their study was associated with the lowest ABI. They concluded that ABI is an accurate and reliable substitute for traditional angiography of peripheral vessels.^26^

Sukhija et al studied the relation between ABI and severity of CAD in patients with peripheral vessel involvement and CAD and reached similar conclusions, i.e. patients with lowest ABI (< 4.0) had the greatest vessel involvement (3 or 4 vessels).^12^ In addition to examining peripheral limb vessels, some studies have assessed the link between ABI and coronaries. Furthermore, studies specifically conducted on men and women have assessed the value of ABI in predicting cardiovascular accidents in the two sexes.

Igarashi et al who used cardiac scanning to identify patients with suspected CAD demonstrated a significant relationship between coronary involvement and low ABI; they suggested that using ABI in addition to other risk factors helps identify high-risk individuals and patients.^27^ The present study confirmed similar results.

A systematic study conducted by Doobay et al investigated the books and articles in which ABI between 0.80 and 0.90 had been used to identify individuals with and without peripheral vessel involvement and had followed these patients to register cardiovascular accidents (death, stroke and myocardial infarction). Of 22 studies, 9 were included in meta-analysis. It was finally concluded that low ABI had high specificity (92.7%) in predicting cardiovascular disease; whereas its sensitivity in doing so was only 16.5%.^28^

A study conducted by Pearson investigated the relationship between low ABI and peripheral vessel disease, cardiovascular disease and other risk factors in women; it demonstrated a special relationship between low ABI and stenosis of coronary vessels, and highlighted ABI as an accurate means for swift identification of atherosclerotic disease.^29^

Imanishi et al studied the value of ABI in predicting CAD in men and concluded that ABI is an independent predictor of risk in men with a predictive value of 8.81% when associated with chest pain, and 71.7% without it.^30^

## Conclusion

In light of earlier studies and results of the present study, we conclude that ABI is an inexpensive and suitable means of estimating peripheral vessel involvement and identifying individuals at high risk of coronary involvement.

We propose that a cohort study be conducted to examine the relationship between ABI and risk of cardiovascular disease, in order to assess the predictive value of this finding.
